# Supplementary material for: Complex‐centric proteome profiling by SEC‐SWATH‐MS
Source: Mol Syst Biol. 2019 Jan 14;15(1):e8438. doi: 10.15252/msb.20188438 (PMC6346213; doi:10.15252/msb.20188438)
Supplement: Supplementary file 7 — Dataset EV6 [file MSB-15-e8438-s007.zip › feature_plots_bioplex/Q9NZ43.pdf]

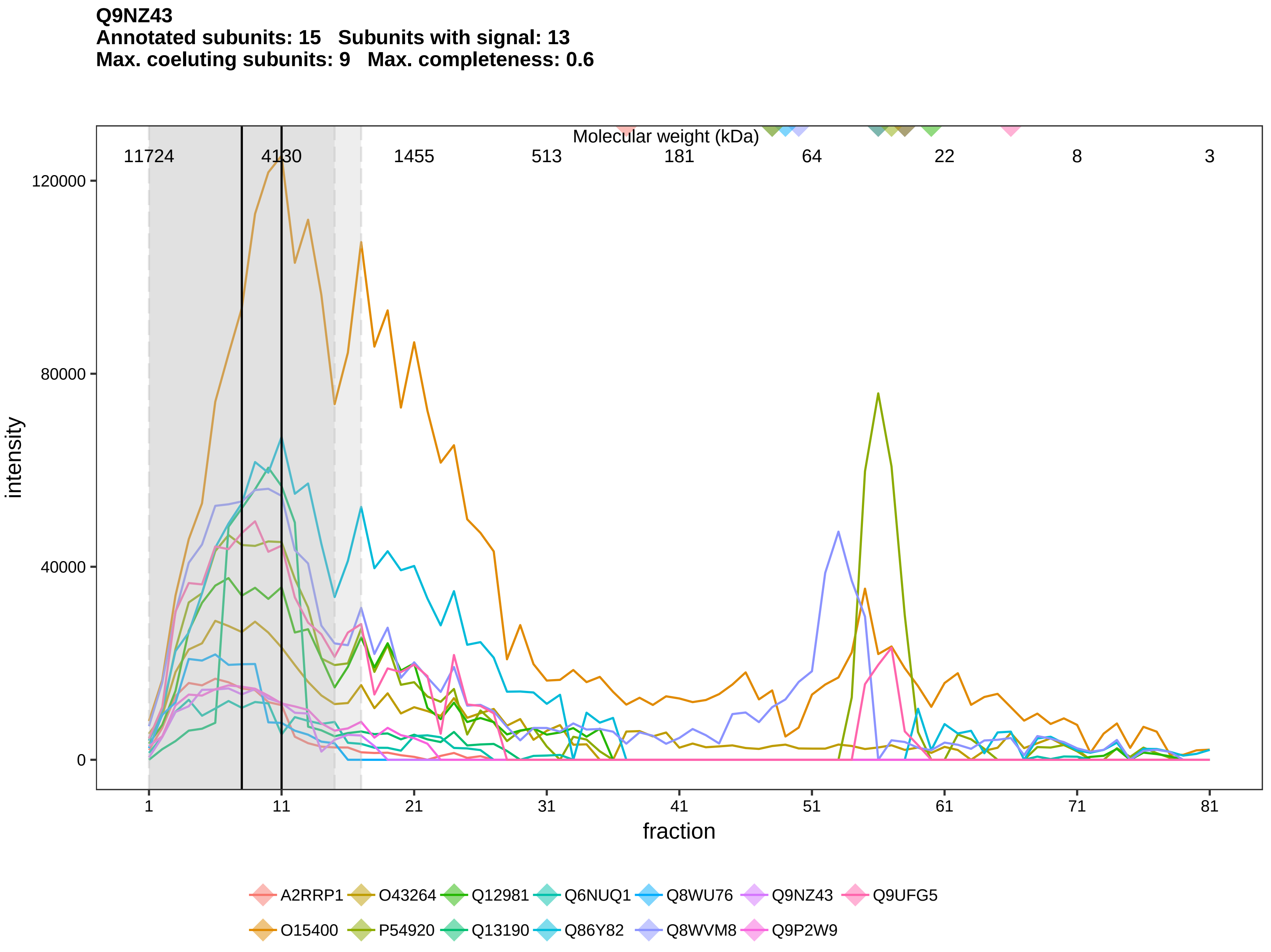

Q9NZ43
Annotated subunits: 15 Subunits with signal: 13
Max. coeluting subunits: 9 Max. completeness: 0.6
Molecular weight (kDa)
11724
4130
1455
513
181
64
22
8
3
120000
80000
40000
0
intensity
1
11
21
31
41
51
61
71
81
fraction
A2RRP1
O43264
Q12981
Q6NUQ1
Q8WU76
Q9NZ43
Q9UFG5
O15400
P54920
Q13190
Q86Y82
Q8WVM8
Q9P2W9
